# Supplementary material for: Oxidized mitochondrial DNA induces gasdermin D oligomerization in systemic lupus erythematosus
Source: Nat Commun. 2023 Feb 16;14:872. doi: 10.1038/s41467-023-36522-z (PMC9935630; doi:10.1038/s41467-023-36522-z)
Supplement: Supplementary file 1 — Supplementary information [file 41467_2023_36522_MOESM1_ESM.pdf]

Oxidized mitochondrial DNA induces gasdermin D oligomerization in systemic lupus erythematosus

Authors: Naijun Miao[1,#], Zhuning Wang[1,#], Qinlan Wang[1], Hongyan Xie[2], Ninghao Yang[2], Yanzhe Wang[3], Jin Wang[1], Haixia Kang[1], Wenjuan Bai[1], Yuanyuan Wang[1], Rui He[1], Kepeng Yan[1], Yang Wang[1], Qiongyi Hu[4], Zhaoyuan Liu[1], Fubin Li[1], Feng Wang[1], Florent Ginhoux[5], Xiaoling Zhang[6], Jianyong Yin[7*], Limin Lu[2*], and Jing Wang[1*]

Affiliations:

[1]Center for Immune-related Diseases at Shanghai Institute of Immunology, Ruijin Hospital, Shanghai Jiao Tong University School of Medicine, Shanghai, China

[2]Department of Physiology and Pathophysiology, School of Basic Medicine Science, Fudan University, Shanghai, China

[3]Department of Nephrology, Shanghai Tong Ren Hospital, Shanghai Jiao Tong University School of Medicine, China

[4]Department of Rheumatology and Immunology, Ruijin Hospital, Shanghai Jiao Tong University School of Medicine, Shanghai, China

[5]Singapore Immunology Network, Agency for Science, Technology and Research, Singapore 138648, Singapore

[6]Department of Orthopedic Surgery, Xinhua Hospital, Shanghai Jiao Tong University School of Medicine, Shanghai 200092, China

[7]Department of Nephrology, Shanghai Jiao Tong University Affiliated Sixth People's Hospital, Shanghai, China

[#]Theses authors contributed equally: Naijun Miao, Zhuning Wang.

[*]Corresponding author Email: jingwang@shsmu.edu.cn (J.W.); lulimin@shmu.edu.cn (L.L.); yinjianyong09@163.com (J.Y.)

Supplemental Figures

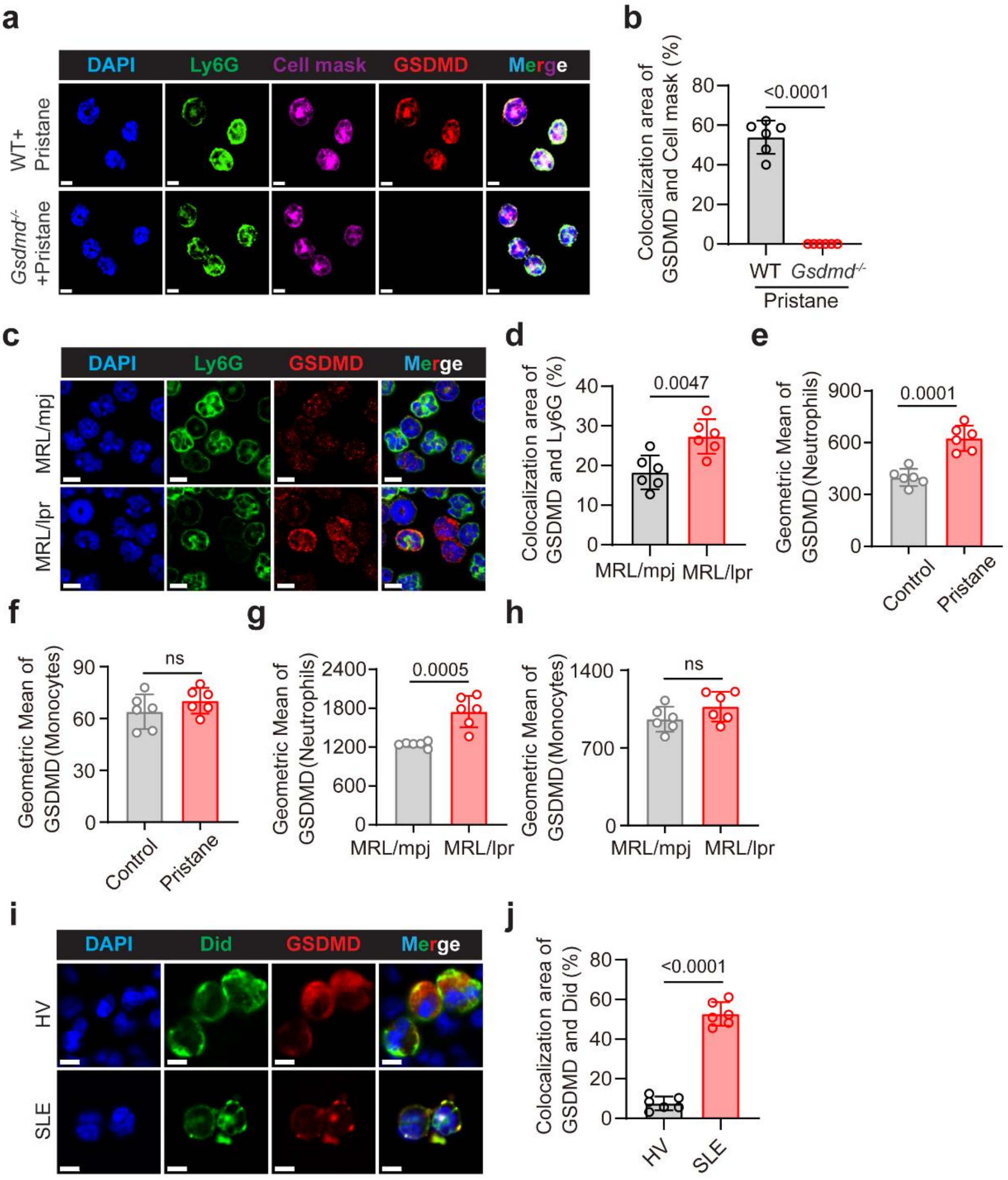

Supplementary Figure 1. The expression of GSDMD on neutrophils from SLE patients and lupus mice. (a) Immunofluorescence staining of Ly6G and GSDMD of BM neutrophils from WT or *Gsdmd*$^{-/-}$ mice after pristane treatment. scale bar, 5 µm. (b) Quantitative analysis of colocalization areas of GSDMD and cell mask. (c) Immunofluorescence staining of Ly6G and GSDMD of BM neutrophils from MRL/mpj or MRL/lpr mice. scale bar, 5 µm. (d) Quantitative analysis of colocalization areas of GSDMD and Did. (e) Quantitative analysis of geometric mean of GSDMD in neutrophils in PIL mice. (f) Quantitative analysis of geometric mean of GSDMD in

monocytes in PIL mice. (g) Quantitative analysis of geometric mean of GSDMD in neutrophils in MRL/lpr mice. (h) Quantitative analysis of geometric mean of GSDMD in monocytes in MRL/lpr mice. (i) Immunofluorescence staining of GSDMD and cell membrane dye Did in peripheral blood neutrophils from HV and SLE patients. Scale bar, 4 μm. (j) Quantitative analysis of colocalization areas of GSDMD and Did. 3 healthy donors and 3 SLE patients were used in one experiment, and plots were pooled from two independent experiments using cells from 6 healthy donors and 6 SLE patients. Three mice were used in one experiment, and plots were pooled from two independent experiments using cells from 6 mice (b, d, e, f, g, h). Data are shown as mean ± SD. Significance was examined with unpaired two-sided Student's *t* test (b, d, e, f, g, h, j).

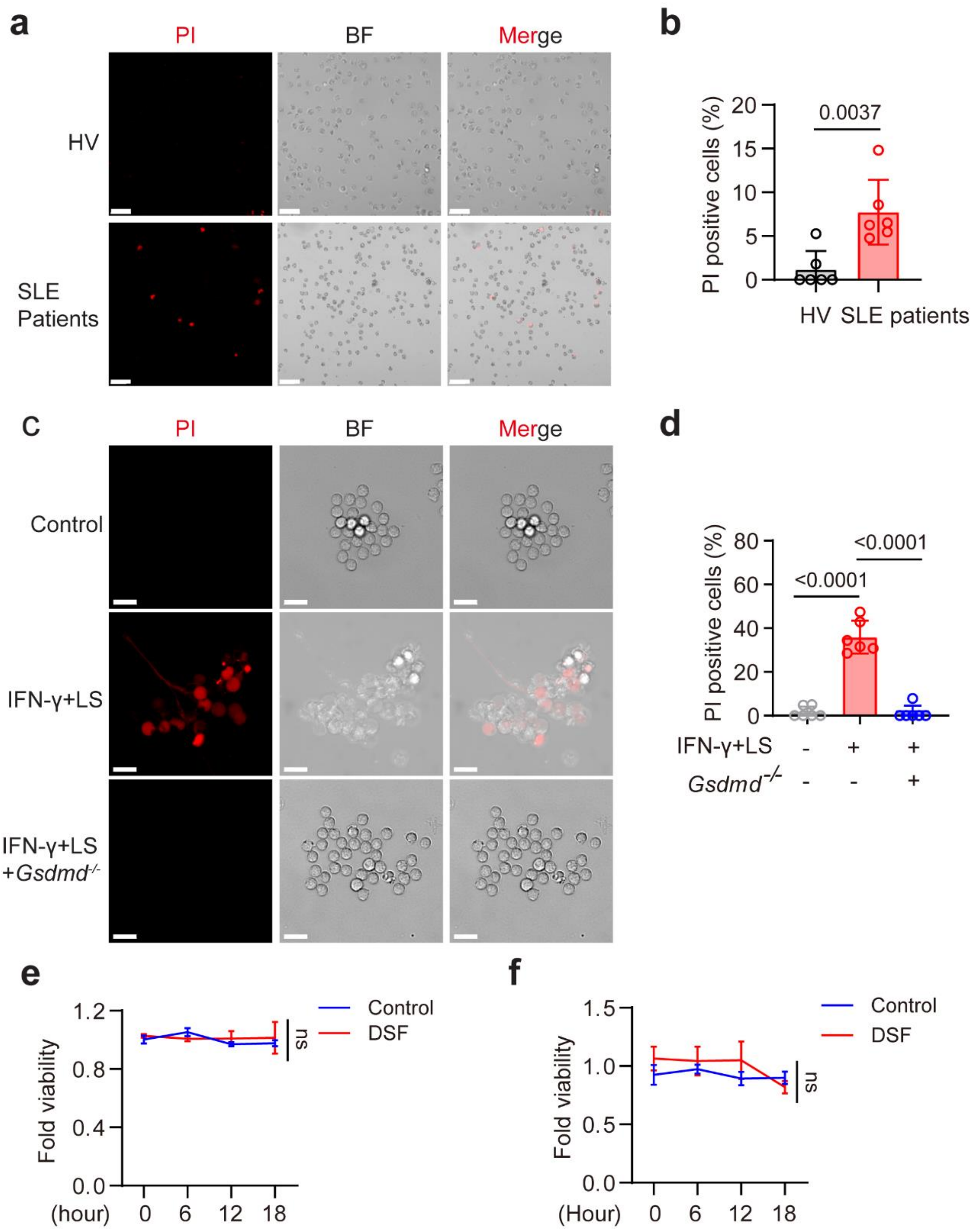

Supplementary Figure 2. The cell death of neutrophils in SLE patients and IFN-γ plus lupus serum treatment. (a) Immunofluorescence staining of PI of peripheral blood neutrophils isolated from HV and SLE patients. Scale bar, 20 μm. (b) Quantitative analysis of percentage of PI staining cells per field of view. 3 healthy donors and 3 SLE patients were used in one experiment, and plots were pooled from two independent experiments using cells from 6 healthy donors and 6 SLE patients. (c) Immunofluorescence staining of PI of BM neutrophils isolated from WT and *Gsdmd*$^{-/-}$

mice. Cells were treated with IFN-γ plus LS. Scale bar, 15 μm. (d) Quantitative analysis of percentage of PI staining cells per field of view. Three mice were used in one experiment, and plots were pooled from two independent experiments using cells from 6 mice. (e) CCK8 analysis of cell viability of BM neutrophils treated with DSF at 5 μM for different times. n = 3 mice, three independent experiments. (f) CCK8 analysis of cell viability of peripheral blood neutrophils from HV. Cells were treated with DSF at 5 μM for different times. n = 3 healthy donors, three independent experiments. Data are shown as mean ± SD. Significance was examined with unpaired two-sided Student's *t* test (b, e, f) or one-way ANOVA test (d).

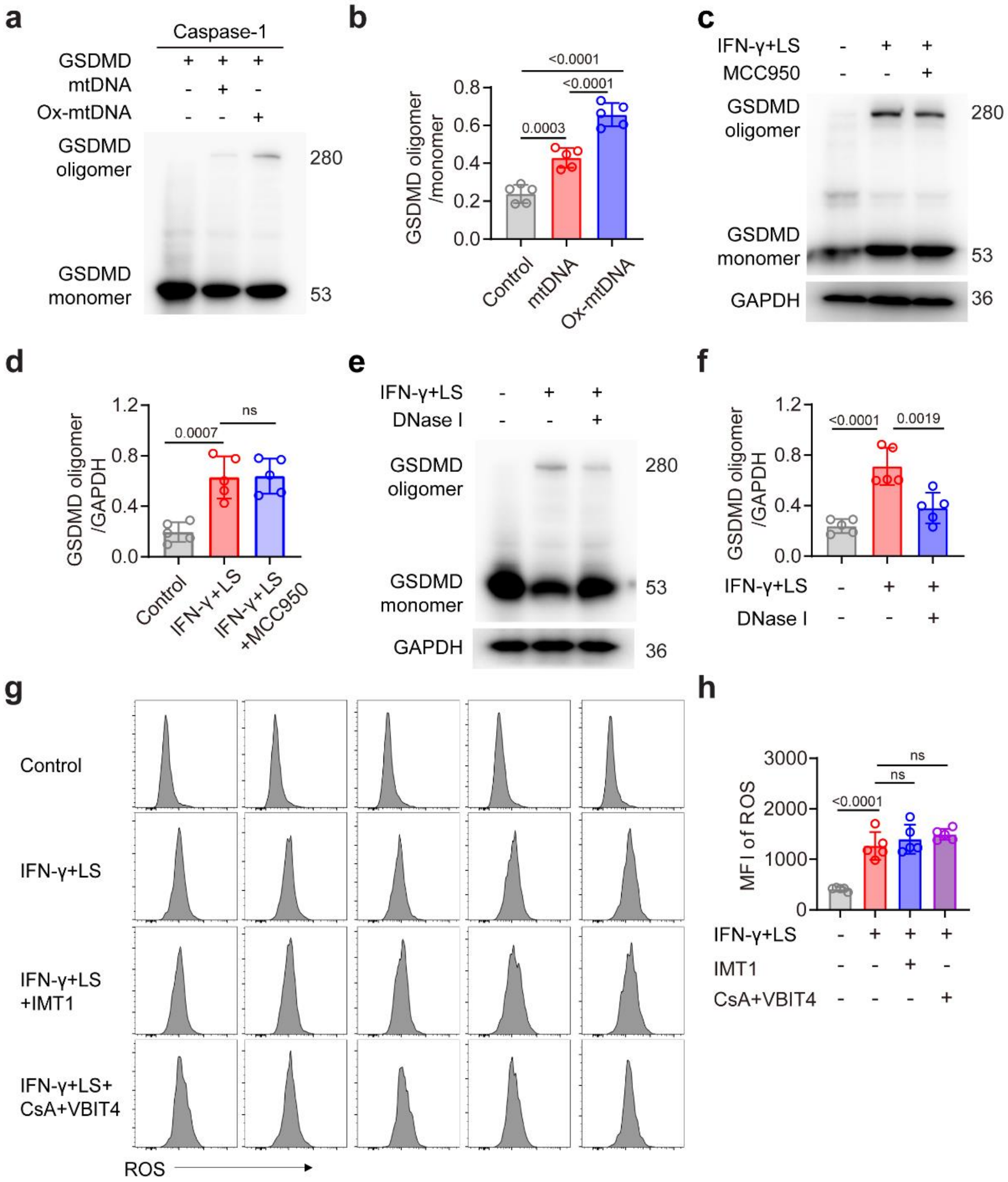

Supplementary Figure 3. MtDNA promotes GSDMD oligomerization. (a) Non-reducing immunoblotting of GSDMD oligomer. Purified GSDMD was incubated with purified active caspase-1 in vitro. The system was then added with mtDNA or Ox-mtDNA (20 nM). (b) Quantitative analysis of GSDMD oligomer/GSDMD monomer. Plots were pooled from five independent experiments. (c) Non-reducing immunoblotting of murine BM neutrophils. Cells were pretreated with MCC950 (7.5 μM). Then the cells were treated with IFN-γ plus LS. (d) Quantitative analysis of GSDMD oligomer/GAPDH. n = 5 mice pooled from 3 independent experiments. (e) Non-reducing immunoblotting of murine BM neutrophils. LS was pretreated with

DNase (0.1 mg/ml). Then the cells were treated with IFN-γ plus LS. (f) Quantitative analysis of GSDMD oligomer/GAPDH. n = 5 mice pooled from 3 independent experiments. (g) Flow analysis of ROS from indicated groups. (h) Quantitative analysis of mean fluorescence intensity (MFI) of ROS. n = 5 mice, 3 independent experiments. Data are shown as mean ± SD. Significance was examined with one-way ANOVA test (b, d, f, h).

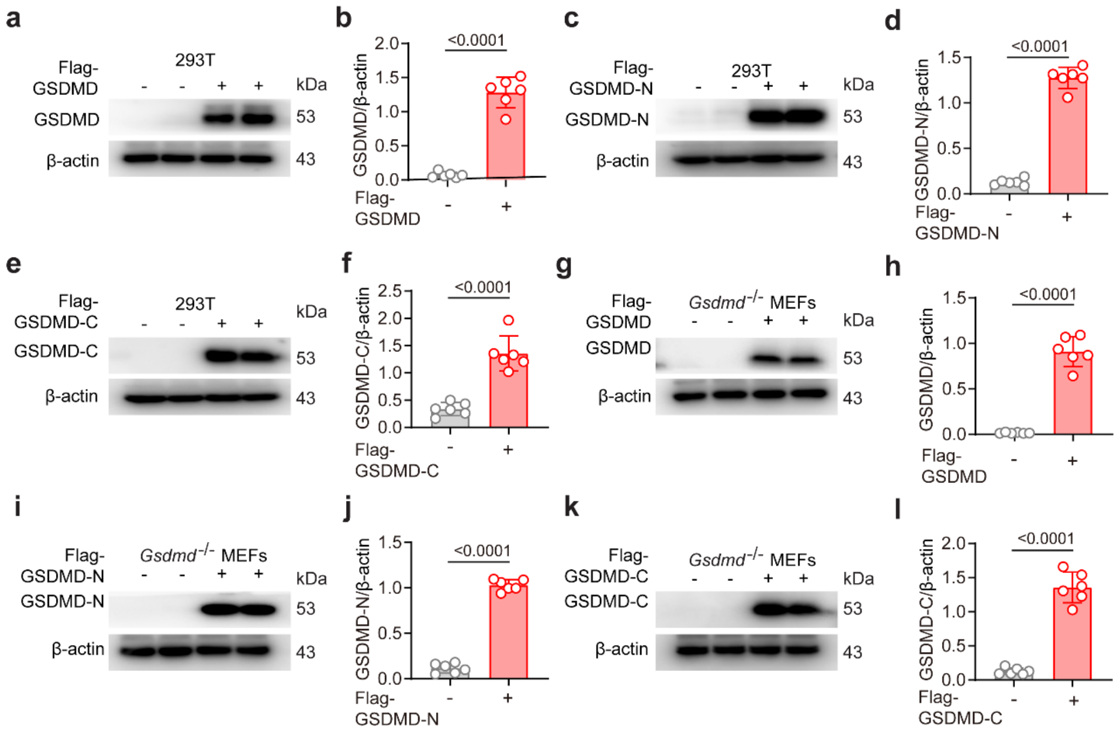

Supplementary Figure 4. Transfection efficiency of GSDMD plasmid in 293T and MEFs. (a-f) 293T cells were transfected with Flag-Full-GSDMD, Flag-GSDMD-N and Flag-GSDMD-C plasmid. Immunoblotting of GSDMD (a), GSDMD-N (c) and GSDMD-C (e). Quantitative analysis of GSDMD (b), GSDMD-N (d) and GSDMD-C (f). (g-l) *Gsdmd*$^{-/-}$ mouse embryonic fibroblasts (MEFs) were transfected with Flag-Full-GSDMD, Flag-GSDMD-N and Flag-GSDMD-C plasmid. Immunoblotting of GSDMD (g), GSDMD-N (i) and GSDMD-C (k). Quantitative analysis of GSDMD (h), GSDMD-N (j) and GSDMD-C (l). n = 6 samples pooled from 3 independent experiments (b, d, f, h, j, l). The immunoblotting samples shown are from the same experiment. Three blots were processed in parallel (b, d, f, h, j, l). Data are shown as mean ± SD. Significance was examined with unpaired two-sided Student's *t* test (b, d, f, h, j, l).

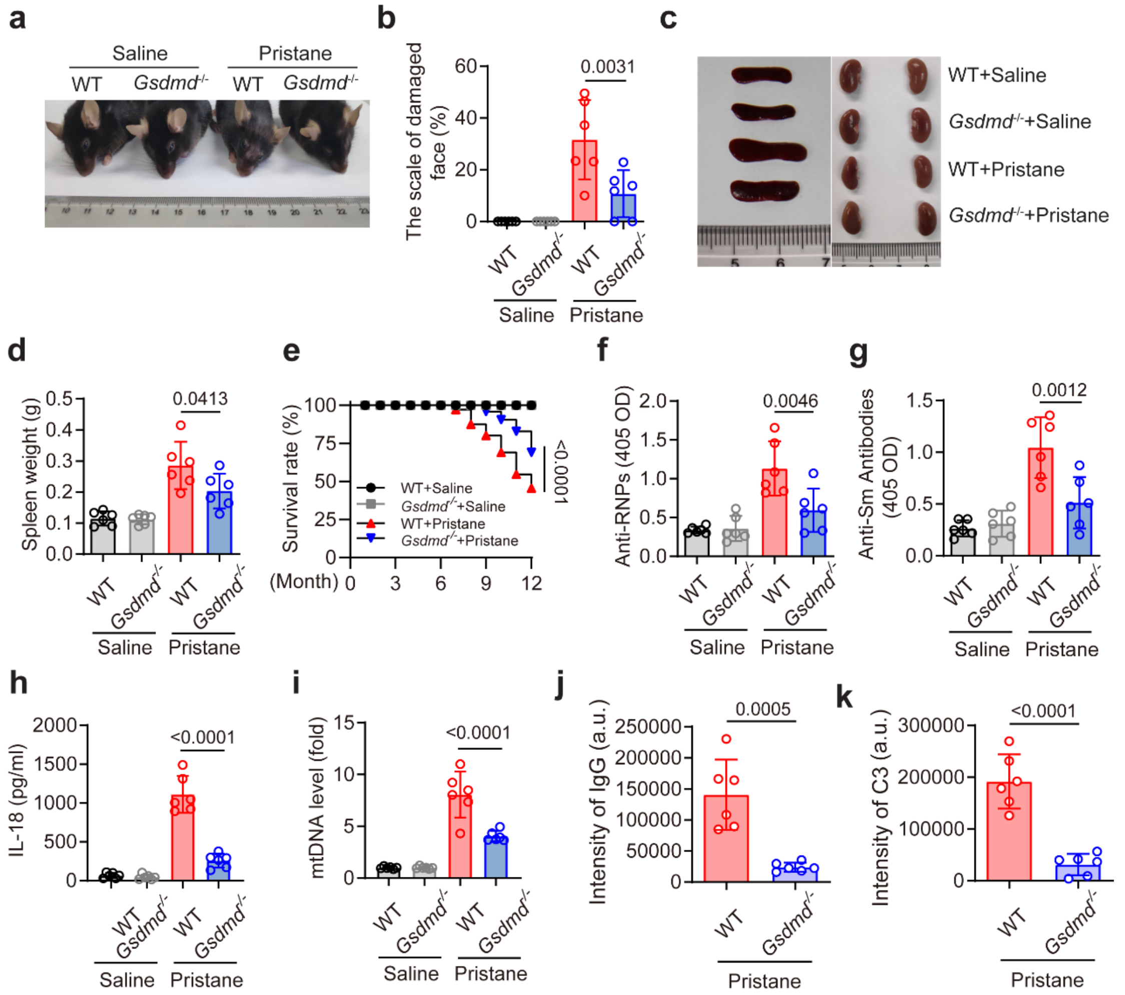

Supplementary Figure 5. GSDMD deficiency ameliorate disease activity in PIL mice. (a) Images of mice from WT + Saline, *Gsdmd*$^{-/-}$ + Saline, WT + Pristane and *Gsdmd*$^{-/-}$ + Pristane group. (b) Quantitative analysis of the areas of facial damage. (c) Images of spleens and kidneys from mice in the indicated groups. (d) Quantification of the weight of spleen in the indicated groups. (e) The survival rate of WT + Saline, *Gsdmd*$^{-/-}$ + Saline, WT + Pristane and *Gsdmd*$^{-/-}$ + Pristane mice. (f-h) ELISA analysis of anti-RNP (f), anti-Sm antibodies (g) and IL-18 (h) in serum from the indicated groups. (i) qPCR analysis of mtDNA from indicated groups. (j and k) Quantitative analysis of the intensity of IgG and C3 from indicated groups. n = 6 mice (b, d-k), two independent experiments (a, c, e-i). Data are shown as mean ± SD. Significance was examined with one-way ANOVA test (b, d, f, g, h, i), Kaplan-Meier (e), unpaired two-sided Student's *t* test (j, k).

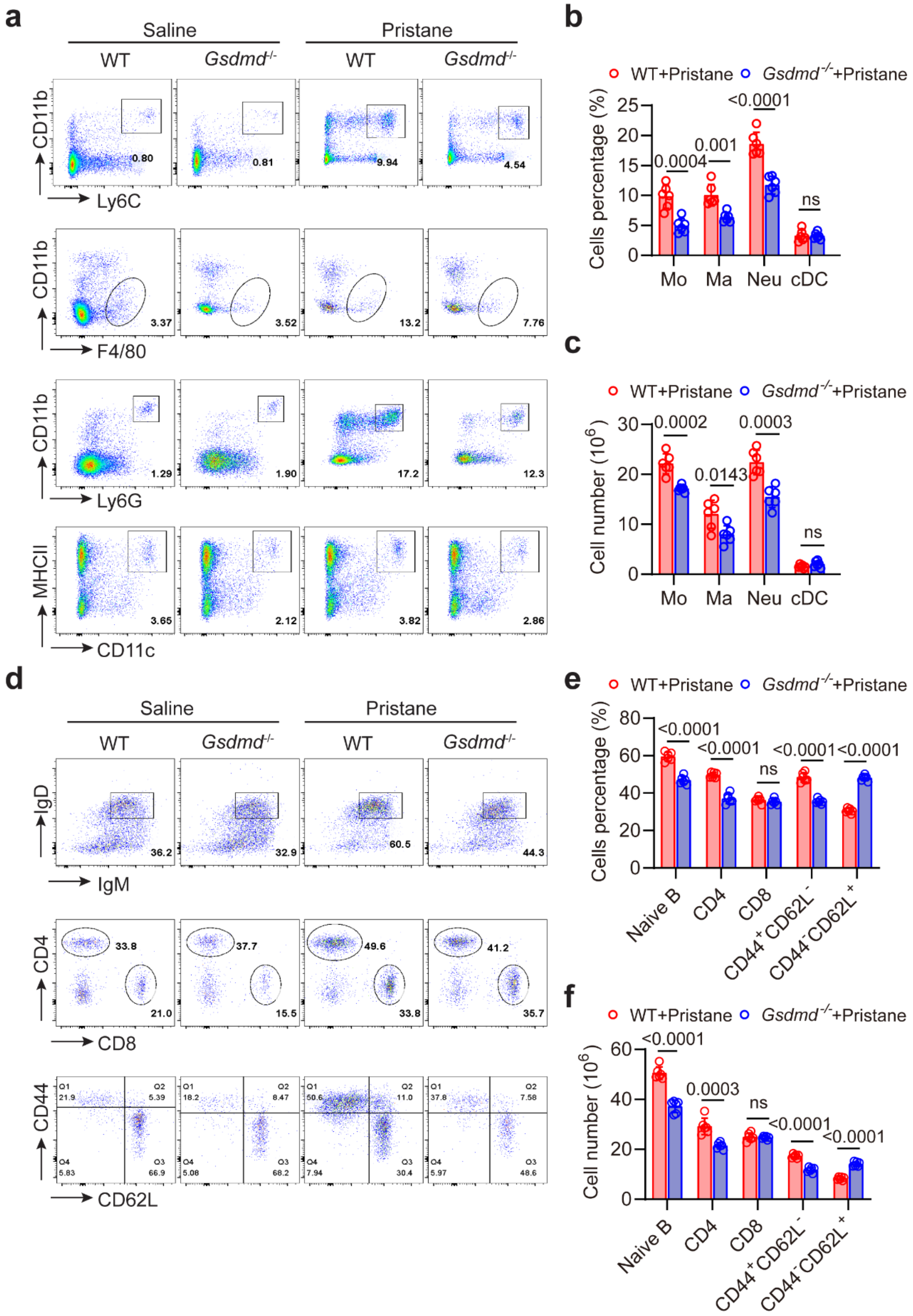

Supplementary Figure 6. The number of myeloid cells and effective T cells are suppressed in *Gsdmd*$^{-/-}$ mice following pristane treatment. (a) Splenic monocytes (CD11b$^+$Ly6G$^-$Ly6C$^+$), macrophages (CD11b$^+$F4/80$^+$), neutrophils (CD11b$^+$Ly6G$^+$), and cDCs (CD11b$^+$CD11c$^+$) were assessed via flow cytometry among all splenic CD45$^+$

cells in WT and *Gsdmd*$^{-/-}$ mice at 7 months following pristane treatment. (b) Quantitative analysis of the relative frequencies of the indicated splenic cell populations in total CD45$^+$ cells. (c) Quantitative analysis of the absolute numbers of the splenic cell populations from indicated groups. (d) Flow cytometry was used to analyze the splenic naïve B cells (B220$^+$IgD$^+$IgM$^+$), CD4$^+$ T cells (CD4$^+$), CD8$^+$ T cells (CD8$^+$), effective T cells (CD44$^+$CD62L$^-$), and naïve T cells (CD44$^-$CD62L$^+$) among total splenic CD45$^+$ cells in WT and *Gsdmd*$^{-/-}$ mice at 7 months after pristane treatment. (e) The relative frequencies of the indicated splenic cell populations in total CD45$^+$ cells were quantified. (f) The absolute numbers of the indicated splenic cell populations were quantified. n = 6 mice (b, c, e, f), two independent experiments (a, d). Data are shown as mean ± SD. Significance was examined with unpaired two-sided Student's *t* test (b, c, e, f).

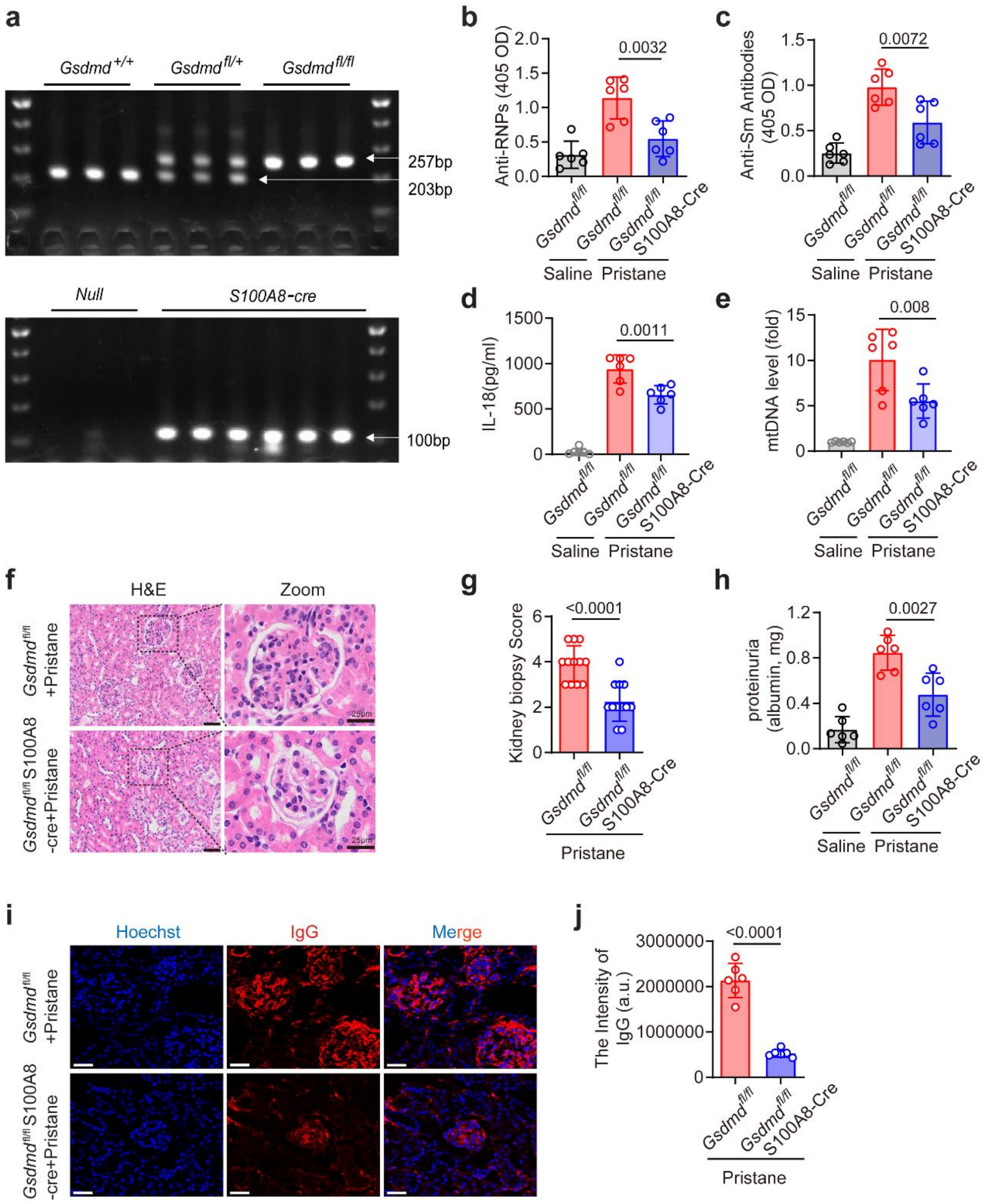

Supplementary Figure 7. Neutrophil GSDMD specific knockout mice ameliorated disease activity in lupus mice. (a) Genetic fingerprinting of *Gsdmd*$^{fl/fl}$ and *S100A8-Cre* mice. (b-d) ELISA analysis of anti-RNPs (b), anti-Sm (c) antibodies and IL-18 (d) in serum from *Gsdmd*$^{fl/fl}$ and *Gsdmd*$^{fl/fl}$*S100A8-Cre* mice were after pristane treatment for 7 months. (e) qPCR analysis of D-loop in serum from the indicated groups. (f) H&E staining of kidney section from *Gsdmd*$^{fl/fl}$ and *Gsdmd*$^{fl/fl}$*S100A8-Cre* mice following pristane treatment for 7 months. Scale bar, 20 μm. (g) Quantitative analysis of kidney biopsy score in *Gsdmd*$^{fl/fl}$ and *Gsdmd*$^{fl/fl}$*S100A8-Cre* mice after pristane treatment. n =

6 mice. One mouse kidney section was quantified by two fields of view. (h) ELISA analysis of Urine albumin levels in *Gsdmd*$^{fl/fl}$ and *Gsdmd*$^{fl/fl}$*S100A8-Cre* mice after pristane treatment. (i) Immunofluorescence staining of IgG in frozen section of kidney from *Gsdmd*$^{fl/fl}$ and *Gsdmd*$^{fl/fl}$*S100A8-Cre* mice following pristane treatment. Scale bar, 25 μm. (j) Quantitative analysis of the intensity of IgG from indicated groups. n = 6 mice (b-e, h, j), two independent experiments (b-e, f, h, j). Data are shown as mean ± SD. Significance was examined with one-way ANOVA test (b, c, d, e, h) or unpaired two-sided Student's *t* test (g, j).

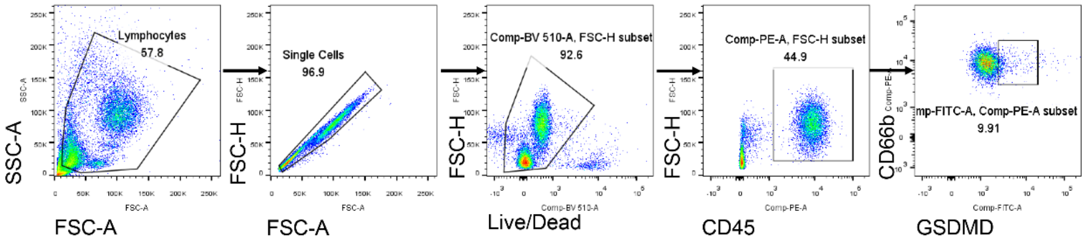

Supplementary Figure 8. The gating strategies for flow cytometry analysis in Figure 1l. Single cells were gated on FSC-A/FSC-H. Live cells were gated on Zombie^{-}/FSC-H. Immune cells were gated on CD45^{+}/FSC-H. The expression of GSDMD in neutrophils was gated on CD66b^{+}GSDMD^{+}.

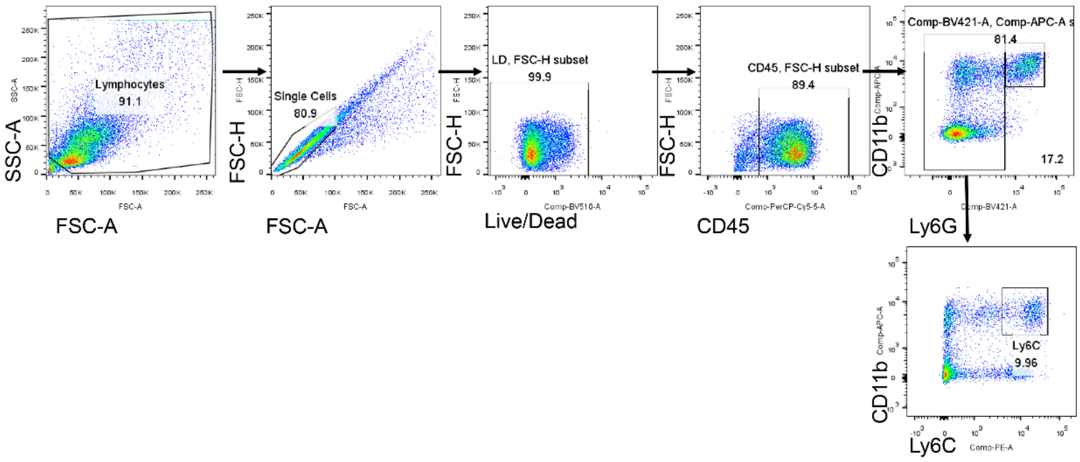

Supplementary Figure 9. The gating strategies for flow cytometry analysis in Supplementary Figure 6a. Single cells were gated on FSC-A/FSC-H. Live cells were gated on Zombie$^-$/FSC-H. Immune cells were gated on CD45$^+$/FSC-H. Neutrophils were gated on CD11b$^+$Ly6G$^+$, Monocytes were gated on CD11b$^+$Ly6G$^-$Ly6C$^+$.

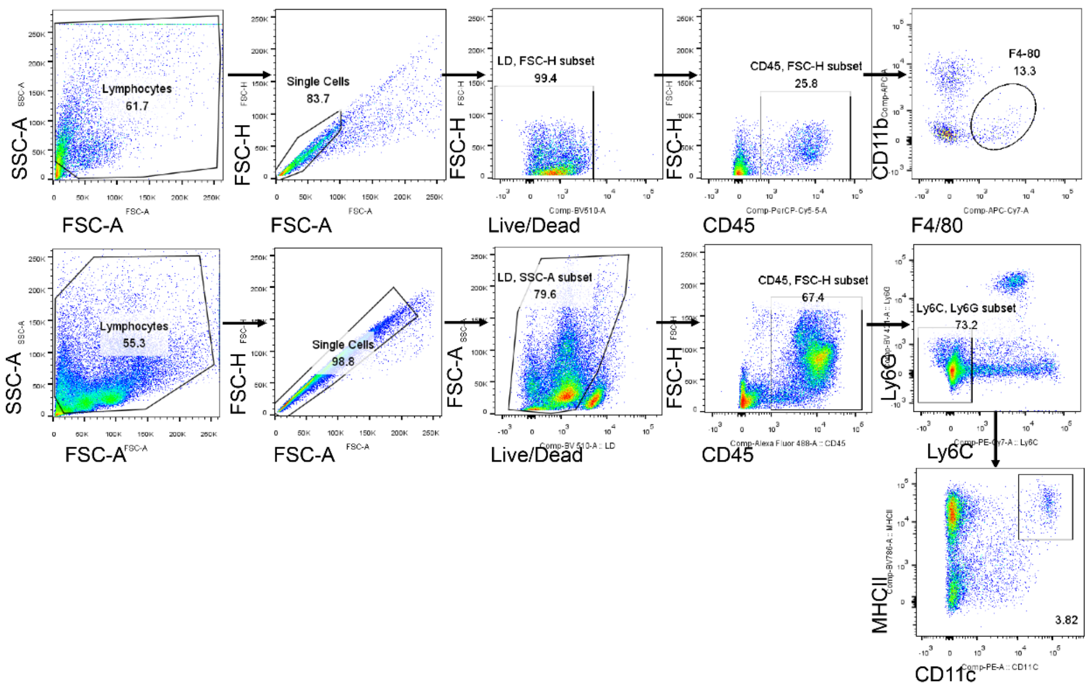

Supplementary Figure 10. The gating strategies for flow cytometry analysis in Supplementary Figure 6a. Single cells were gated on FSC-A/FSC-H. Live cells were gated on Zombie$^-$/FSC-H. Immune cells were gated on CD45$^+$/FSC-H. Macrophages were gated on CD11b$^+$F4/80$^+$. cDC were gated on CD45$^+$/Ly6G$^-$/Ly6C$^-$/CD11c$^+$/MHCII$^+$.

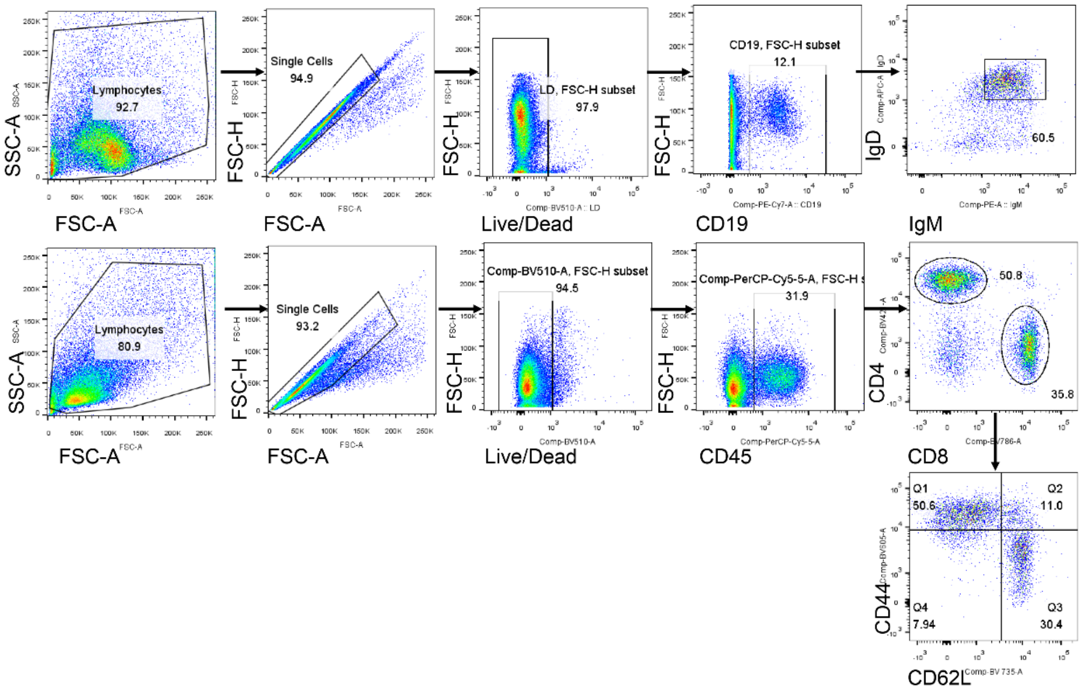

Supplementary Figure 11. The gating strategies for flow cytometry analysis in Supplementary Figure 6d. Single cells were gated on FSC-A/FSC-H. Live cells were gated on Zombie-/FSC-H. Immune cells were gated on $CD45^+$/FSC-H. Naïve B cells were gated on $CD19^+IgM^+IgD^+$. T cells were gated on $CD4^+$ and $CD8^+$ separately. The effective $CD8^+$T cells were gated on $CD62L^-/CD44^+$.

Supplementary Table 1. Demographic data of SLE patients.

Characteristic	SLE patients (n = 34)	Healthy donors (n =10)
Sex (male/female)	8/26	4/6
Age, years (mean ± SD)	43.36 ± 11.27	30.45 ± 9.33
Disease duration, months (mean ± SD)	60.23 ± 23.47	——
SLEDAI score (mean ± SD)	21.23 ± 7.74	——
Anti-ANA (positive/negative)	34/0	——
Proteinuria (positive/negative)	34/0	——

ANA, antinuclear antibody. SLEDAL, systemic lupus erythematosus disease activity index.

Supplementary Table 2. Key resources used in this study.

REAGENT or RESOURCE	DILUTION	SOURCE	IDENTIFIER
Anti-Caspase-1 antibody	1:500	Adipogen	AC-20B-00420-C100
Anti-GSDMD/GSDMD-N antibody (Clone: EPR20859)	1:2000	Abcam	ab219800
Anti-Caspase-11 antibody (Clone: 17D9)	1:500	Sigma	C1354
Anti-GAPDH antibody (Clone: D16H11)	1:5000	Cell signaling technology	5174S
Anti-Serpinb1 antibody	1:500	ThermoFisher	PA5-76875
Anti-caspase-4 antibody	1:1000	Abcam	ab25898
Anti-MPO antibody (Clone: 2D4)	1:200	Abcam	ab90812
Anti-TOMM20 antibody (Clone: EPR15581-54)	1:500	Abcam	ab186735
Anti-8OHdG antibody (Clone: 15A3)	1:500	Abcam	ab62623
Anti-Elastase antibody	1:1000	Abcam	ab21595
PB-Rat anti-mouse (Clone: 1A8)	1:500	Biolegend	127626
APC-Rat anti-mouse/human CD11b (Clone: M1/70)	1:1000	Biolegend	101212
PerCP/Cy5.5-Rat anti-mouse CD45 (Clone: 30-F11)	1:1000	Biolegend	103132
PE/Cyanine7-anti-human CD45 (Clone: 2D1)	1:1000	Biolegend	368531
PE-anti-mouse Ly6C (Clone: HK1.4)	1:1000	Biolegend	128008
PE-anti-human CD66b (Clone: G10F5)	1:500	Biolegend	305106
BV786-Rat anti-mouse MHCII (Clone: M5/114.15.2)	1:1000	Biolegend	107645
PE-anti-mouse CD11c (Clone: N418)	1:1000	Biolegend	117308
APC-Cyanine7-anti-mouse IgD (Clone: 11-26c.2a)	1:500	Biolegend	405715
APC-anti-mouse IgM (Clone: RMM-1)	1:500	Biolegend	406509
BV421-anti-mouse CD4 (Clone: RM4-5)	1:500	Biolegend	100544
BV786-anti-mouse CD8 (Clone: 53-6.7)	1:1000	Biolegend	100750
PE-cy7-Rat anti-mouse CD44 (Clone: IM7)	1:1000	Biolegend	103029
FITC-CD62L-Rat anti-mouse CD62L (Clone: MEL-14)	1:500	Biolegend	104405

Supplementary Table 3. Primers used in this study.

Gene	Sequence (5’ > 3’)	Species
ND1	Forward: AACACTTATTACAACCCAAGAACA<br>Reverse: TCATATTATGGCTATGGGTCAGG	Mouse
ND2	Forward: TCCTATCACCCTTGCCATCAT<br>Reverse: CTGCTTCAGTTGATCGTGGG	Mouse
Gapdh	Forward: TGCACCACCAACTGCTTAGC<br>Reverse: GGCATGGACTGTGGTCATGAG	Mouse
Hrpt1	Forward: TGAAGTACTCATTATAGTCAAGGGCA<br>Reverse: CTGGTGAAAAGGACCTCTCG	Mouse
mtDNA primer 1	Forward: CACCCAAGAACAGGGTTTGT<br>Reverse: TGGCCATGGGTATGTTGTTAA	Mouse
mtDNA primer 2	Forward: CTATCACCCTATTAACCACTCA<br>Reverse: TTCGCCTGTAATATTGAACGTA	Mouse
D-loop	Forward: TCCTCCGTGAAACCAACAA<br>Reverse: AGCGAGAAGAGGGGCATT	Mouse
Gsdmd	Forward: ATGAGGTGCCTCCACAACTTCC<br>Reverse: CCAGTTCCTTGGAGATGGTCTC	Human
Gapdh	Forward: AGGCAACTAGGATGGTGTGG<br>Reverse: TTGATTTTGGAGGGATCTCG	Human

Supplementary Movie 1. Neutrophil motion trails in WT mice. Representative two-photon intravital imaging of neutrophils in the kidneys of WT mice. Texas Red-dextran 70 kD (10 μg/mouse) and AF488-anti-mouse ly6G (2.5 μg/mouse) were administered intravenously 10 minutes before imaging (n = 3). Scale bar, 50 μm.

Supplementary Movie 2. The glomeruli of PIL mice exhibit increased neutrophil infiltration. Representative two-photon intravital imaging of neutrophils in the kidneys of PIL mice. Texas Red-dextran 70 kD (10 μg/mouse) and AF488 anti-mouse ly6G (2.5 μg/mouse) were administered intravenously 10 minutes before imaging (n = 3). Scale bar, 50 μm.

Supplementary Movie 3. The release of extracellular DNA as "punctate particles" from neutrophils in the glomeruli of PIL mice. Representative two-photon imaging of neutrophil extracellular DNA release in Ms4a3-tdTomato mice following pristane treatment for 7 months. Mice were intravenously administered with Qtracker 655 and Sytox Green for 10 minutes before imaging (n = 3). Scale bar, 20 μm.

Supplementary Movie 4. The release of extracellular DNA as "NETs-like structures" from neutrophils in the glomeruli of PIL mice. Representative two-photon imaging of neutrophil extracellular DNA release in Ms4a3-tdTomato mice following pristane treatment for 7 months. Mice were intravenously administered with Qtracker 655 and Sytox Green for 10 minutes before imaging (n = 3). Scale bar, 20 μm.

Supplementary Movie 5. Sytox Green^{+} neutrophil accumulation within the glomerular capillaries of wild type mice. Representative two-photon intravital imaging of the kidneys of pristane-treated WT mice. Qtracker 655, Sytox Green, and PE-anti-mouse Ly6G were administered intravenously 10 minutes before imaging (n = 3). Scale bar, 20 μm.

Supplementary Movie 6. Sytox Green^{+} neutrophil accumulation within the glomerular capillaries of *Gsdmd*$^{-/-}$ mice. Representative two-photon intravital imaging of the kidneys of pristane-treated *Gsdmd*$^{-/-}$ mice. Qtracker 655, Sytox Green, and PE-anti-mouse Ly6G were administered intravenously 10 minutes before imaging (n = 3). Scale bar, 50 μm.
