# Supplementary material for: Oxidized mitochondrial DNA induces gasdermin D oligomerization in systemic lupus erythematosus
Source: Nat Commun. 2023 Feb 16;14:872. doi: 10.1038/s41467-023-36522-z (PMC9935630; doi:10.1038/s41467-023-36522-z)
Supplement: Supplementary file 4 — Description of additional supplementary files [file 41467_2023_36522_MOESM4_ESM.docx]

**Description of Additional Supplementary Files**

Supplementary Movie 1

Description: Representative two-photon intravital imaging of neutrophils in the kidneys of WT mice. Texas Red-dextran 70 kD (10 μg/mouse) and AF488-anti-mouse ly6G (2.5 μg/mouse) were administered intravenously 10 minutes before imaging (n = 3). Scale bar, 50 μm.

Supplementary Movie 2

Description: Representative two-photon intravital imaging of neutrophils in the kidneys of PIL mice. Texas Red-dextran 70 kD (10 μg/mouse) and AF488 anti-mouse ly6G (2.5 μg/mouse) were administered intravenously 10 minutes before imaging (n = 3). Scale bar, 50 μm.

Supplementary Movie 3

Description: Representative two-photon imaging of neutrophil extracellular DNA release in Ms4a3-tdTomato mice following pristane treatment for 7 months. Mice were intravenously administered with Qtracker 655 and Sytox Green for 10 minutes before imaging (n = 3). Scale bar, 20 μm.

Supplementary Movie 4

Description: Representative two-photon imaging of neutrophil extracellular DNA release in Ms4a3-tdTomato mice following pristane treatment for 7 months. Mice were intravenously administered with Qtracker 655 and Sytox Green for 10 minutes before imaging (n = 3). Scale bar, 20 μm.

Supplementary Movie 5

Description: Representative two-photon intravital imaging of the kidneys of pristane-treated WT mice. Qtracker 655, Sytox Green, and PE-anti-mouse Ly6G were administered intravenously 10 minutes before imaging (n = 3). Scale bar, 20 μm.

Supplementary Movie 6

Description: Representative two-photon intravital imaging of the kidneys of pristane-treated *Gsdmd^-/-^* mice. Qtracker 655, Sytox Green, and PE-anti-mouse Ly6G were administered intravenously 10 minutes before imaging (n = 3). Scale bar, 50 μm.
